# Revision of a Fractured Uncemented Revision Stem Using a Custom Designed Punch and Retrograde Through-Knee Approach

**DOI:** 10.1155/2015/485729

**Published:** 2015-02-22

**Authors:** P. J. Nasr, G. S. Keene

**Affiliations:** Addenbrooke's Hospital, Cambridge Hospitals NHS Foundation Trust, Cambridge CB2 0QQ, UK

## Abstract

We report a unique case of a fractured modular cobalt chromium connection taper Revitan (Zimmer, Warsaw, IN) revision prosthesis. Macroscopic examination revealed a fracture at the diaphyseal-metaphyseal junction of this modular component. This report highlights that fractures can still occur with modern modular prostheses. We are not aware of any published failures of the Revitan revision prosthesis. We also describe a unique method of retrieval for a broken well fixed uncemented femoral stem, using a custom designed extraction instrument via a through-knee approach.

## 1. Introduction

The success of revision total hip replacement surgery is dependent on three main factors: the design of the prosthesis, the surgical technique, and the patient. Revisions can either be cemented or uncemented and one mode of fixation uses the press-fit principle to achieve stability. Cementless stems can achieve stability by gaining fixation in the proximal or distal femur. The advantage of gaining stability in the mid-femur is that bone stock is often superior in the revision setting. Fixation can occur via several methods, including extensively porous coated stems, as well as fluted, tapered, grit blasted stems. One such example of an uncemented tapered, fluted, modular implant is the Revitan stem (Zimmer, Warsaw, IN).

The Revitan modular revision system is based on the concept of press-fit anchorage and the connection technology is designed to be flexible and reliable [1]. The proximal components are available in a cylindrical or conical design, both with lengths ranging from 55 to 105 mm in 10 mm increments. The distal and proximal components are designed to be combined to achieve good fit and optimisation of leg length. The slim neck is designed to increase range of motion and also decrease the risk of impingement. The offset is 44 mm to allow good gluteal muscle function. The stem is ribbed to allow a press-fit into the proximal femur. The proximal and distal components are attached via the Revitan connection taper, which was introduced in 1990 and has been implanted in over 20,000 cases. The taper is made of forged cobalt chromium (CoCr) alloy (Protasul 21 WF) which is of a higher strength than other materials such as titanium alloy. The manufacturer describes 4 zones of the taper with the maximal stress of the bending forces being concentrated on the section with the smallest diameter, which is claimed to prevent the formation of metal debris by not allowing the proximal and distal components to contact.

## 2. Case History

A 58-year-old male patient underwent a second revision left total hip replacement for loosening years previously—the index revision. His BMI at the time of the index revision procedure was 29. The previous revision Charnley total hip replacement was undertaken through an anterolateral approach and the index revision procedures was undertaken through a posterior approach.

During the index revision procedure a straight Revitan stem (Zimmer) was implanted with a cylindrical 55 mm proximal component and a 22 × 200 mm diaphyseal component attached via the Revitan connection taper. A 32/0 ceramic head was inserted. A 52/32 Exeter Contemporary (Stryker, Warsaw, IN) acetabular component was cemented using Palacos R, having reconstructed the bone loss in the acetabulum using a Contour reinforcement ring (Smith & Nephew, London, UK). The hip was stable at the end of the procedure with equal leg lengths. There were no immediate postoperative complications. Subsequent early followup was unremarkable.

**Figure 1:**
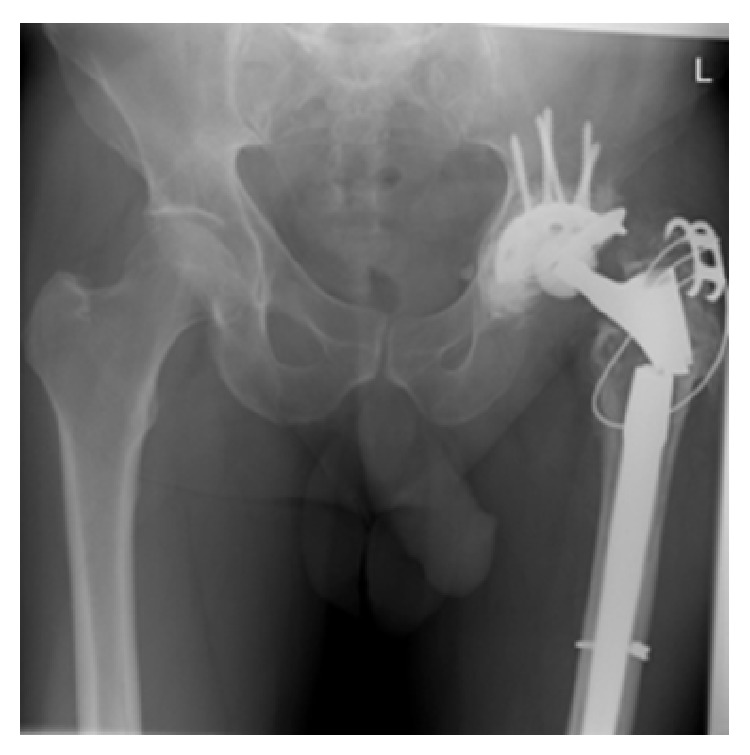
Plain AP pelvic radiograph of broken Revitan stem.

Four years later, the patient presented with pain in his left hip and some weakness. Clinical examination revealed new shortening of his leg associated with pain on attempted straight leg raising. His BMI was unchanged. Radiographic examination showed a fracture around the Revitan connection taper, which was confirmed following explant of the prosthesis (Figure 1). There was no evidence of infection.

A decision was made to revise the prosthesis and a unique operative plan to retrieve the distal part of the broken femoral stem using a custom made instrument inserted via the knee in a retrograde fashion, rather than split the femur to retrieve the prosthesis and then use a longer stem. The 14 mm diameter instrument was manufactured by the surgical devices unit at our University Hospital, as a 500 × 14 mm long straight punch, with the end shaped in a concave fashion to engage the tip of the femoral stem.

**Figure 2:**
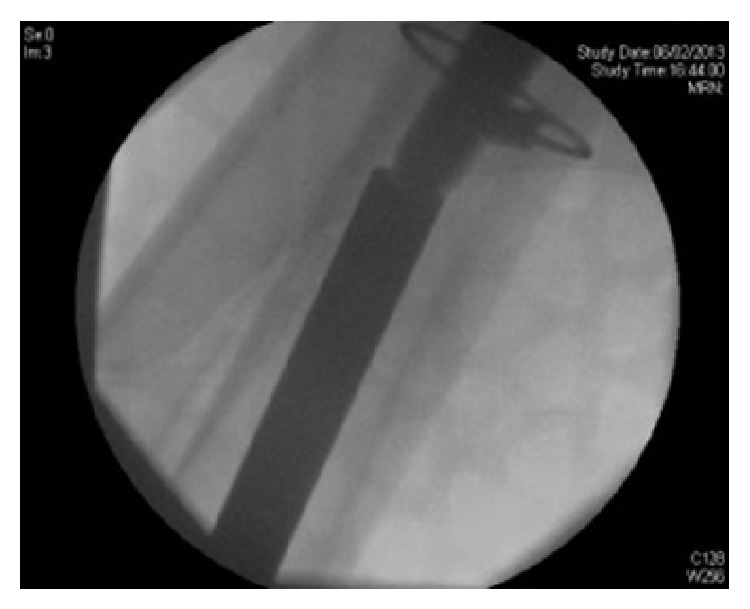
Intraoperative image, intensifier image of custom made instrument contacting tip of Revitan femoral component.

**Figure 3:**
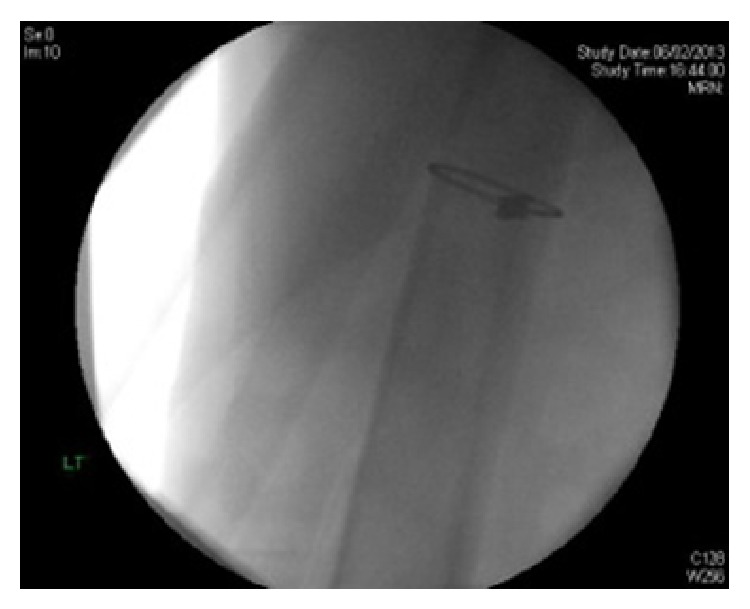
Intraoperative Image Intensifier image of femoral diaphysis after removal of Revitan femoral component, showing no evidence of fracture or loss of bone stock.

A posterior approach was used to the hip. The stem could not be retrieved from the area around the greater trochanter. Attention was then made to retrieval of the well fixed diaphyseal portion of the broken stem. A midline incision was made over the knee and a medial parapatellar approach performed. A 16 mm drill was used to gain entry to the distal femoral metaphysis and our custom made instrument inserted retrograde to punch the well fixed femoral component towards the proximal femur. Image intensifier guidance was used throughout the procedure (Figure 2). The stem was disengaged and then retrieved with ease, without any fracture occurring or loss of the femoral bone stock (Figure 3).

**Figure 4:**
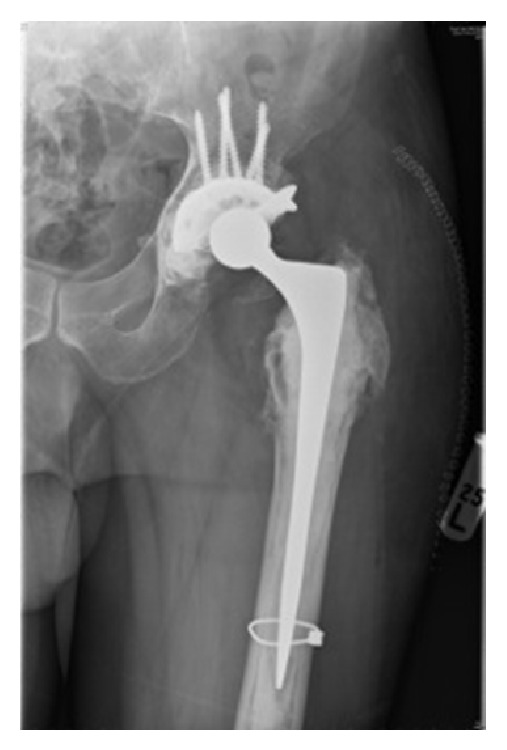
Postoperative radiograph of cemented CPT size 4 extended offset stem.

Thereafter, the stem was revised to a cemented CPT size 4 extended offset 200 mm stem (Zimmer, Warsaw, IN) (Figure 4). There were no immediate postoperative complications and the patient was discharged on day 5. Subsequent followup has been unremarkable with no symptoms of knee pain and a well functioning revision hip replacement.

**Figure 5:**
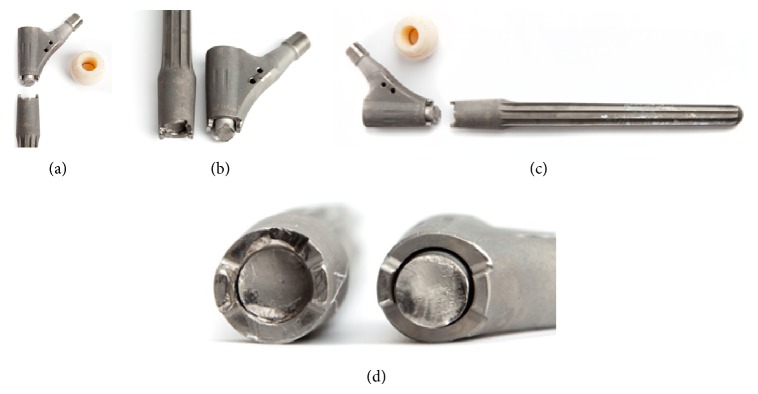
Photographs of retrieved Revitan stem.

Analysis of the explanted stem showed that it had fractured at the connection taper (Figure 5).

## 3. Discussion

To our knowledge this is the first reported fracture of the Revitan revision prosthesis.

Numerous factors have been shown to increase the risk of femoral component fracture in the setting total hip replacement. Patient-related factors include male gender, increased weight, increased height, high-activity levels, bilateral hip disease, lumbar spine disease, and the presence of bilateral total hip replacements (THRs) [2]. Surgical factors include varus stem orientation, poor proximal fixation coupled with rigid distal fixation leading to cantilever bending/fatigue, undersized femoral component, and poor proximal bone support shown by absence of the calcar [3]. Factors associated with the prosthesis include improper material selection, manufacturing or metallurgic defects, and design flaws leading to stress risers [4].

The cross-sectional shape of a press fit, uncemented stem is important for rotational stability. The Revitan stem has cutting flutes which decrease rotational forces acting throughout the femur and also may aid regeneration of bone by allowing revascularization. There is ventrodorsal flattening, which increases with the diameter of the implant, to give elasticity and prevent stress-shielding. The original Wagner stem, which had a similar fluted, tapered design, has been reported to have good results in the literature [5].

Any tapered uncemented stem will achieve stability at quite unpredictable locations; hence stem modularity allows varying lengths of the proximal body to optimize leg length, femoral version, offset, and ultimately stability of the hip. With modular stems, a great amount of research has gone into the engineering of the connection taper.

This study highlights a failure that led to a stress riser forming in an implant that was well fixed distally in the femoral diaphysis. Modular systems do increase the risk of corrosion at the taper [6, 7]. The design of the Revitan taper is such that it is designed to have the maximal stress of the bending forces concentrated on a section with the smallest diameter to decrease the risk of wear debris being caused by contact between the proximal and distal components. We postulate that the cause of this failure was in part due to the large stresses being concentrated on such a narrow taper.
